# How volunteer engagement experiences relate to the social responsibility of university students: the roles of emotional intelligence and relational embeddedness

**DOI:** 10.3389/fpsyg.2026.1759354

**Published:** 2026-04-29

**Authors:** Yixin Jian, Lingjun Ou, Xiaoqing Gao

**Affiliations:** 1 College of Social Development, College of Tourism and Aviation Management, Hunan Women's University, Changsha, China; 2 College of Educational Science, Hunan Normal University, Changsha, China

**Keywords:** emotional experience, emotional intelligence, relational embeddedness, social responsibility of university students, volunteer engagement

## Abstract

There is abundant research on how to enhance the social responsibility of university students, most of which attribute the development to observational learning from role models or to value comparisons in social exchanges. Different from these perspectives, this study posits that the expansion–construction of emotional experiences in social interactions is a key mechanism, and Volunteer engagement is one of the contexts that can provide rich Emotional experience. A grouped comparative analysis with 5,088 university student volunteers found no significant difference in social responsibility of university students between groups defined by Volunteer Service Participation (i.e., having participated vs. not). In other words, participating in Volunteer engagement alone does not directly increase students' social responsibility. Further cross-sectional survey with group comparison (*N* = 4,498) and SEM mediation in a volunteer subgroup showed that the intensity of *Emotional experience during Volunteer engagement* is positively and significantly associated with social responsibility, and that emotional intelligence and relational embeddedness play a dual mediating role between them. This provides an affective activation pathway for enhancing students' social responsibility through Volunteer engagement. Based on these findings, universities can take action on three fronts: (1) design and cultivate emotionally engaging volunteer experiences to enhance affective arousal mechanisms; (2) integrate emotional intelligence development into volunteer service frameworks; and (3) strengthen relational embedding in volunteer activities to foster long-term social responsibility.

## 1 Introduction

The sense of social responsibility among University students refers to their cognition, emotions, and behavioral tendencies concerning the duties and obligations they should fulfill as members of society. It is regarded as a relatively stable psychological quality (Huang et al., 2016). The concept of social responsibility is broad and encompasses a wide range of factors. The concept of obligations encompasses a wide range of commitments, including those toward oneself, other individuals, collective entities, the state, the nation, all of humanity, and even nature. These obligations collectively address all responsibilities that are deemed conducive to the advancement of human socialization (Lu and Koehn, 2015).

Extant research on social responsibility has followed two theoretical trajectories: social learning theory and social exchange theory. The former posits that individuals acquire social behaviors and values by observing, learning from, and imitating role models (Tekleab et al., 2021). Within social contexts, when individuals witness socially responsible behavior being rewarded or praised, they are more likely to imitate such conduct and eventually internalize it as part of their own sense of social responsibility. The latter emphasizes that social responsibility appears through value comparisons in the process of social interaction. In balancing costs and benefits, individuals are more inclined to behave responsibly when the perceived returns of assuming responsibility outweigh the costs (Bénabou and Tirole, 2010). Nevertheless, empirical evidence suggests that the relationship between role-model learning and the development of social responsibility is not strictly linear, indicating potential misalignments (Baden, 2014). This poses new challenges to the explanatory power of social learning theory and opens up space for reflection. Moreover, scholars have argued that human behavior is shaped not only by rational calculations but also by emotions, social norms, and moral considerations. Consequently, the assumption of the “rational economic actor” within social exchange theory fails to adequately account for altruistic behavior (Tian and Ou, 2023), thereby limiting its explanatory power in exploring the mechanisms underlying the cultivation of social responsibility among university students.

When examining the mechanisms underlying the formation of social responsibility, the role of emotion cannot be overlooked. Its philosophical and ethical roots trace back to Aristotle (Symons and VanderWeele, 2024). Ethical virtue is not merely the outcome of rational choice but also concerns the appropriate expression of emotions and the cultivation of habits. Its essence is fundamentally “the quality of the emotions” (Intellectualism in Aristotle, 1978). This perspective, which regards emotions as a core element in the cultivation of virtue, provides a classical theoretical foundation for understanding the emotional basis of social responsibility. The formation of responsibility is not the abstract internalization of norms detached from emotion, but rather a process through which individuals gradually shape appropriate emotional responses and inclinations through practice while interacting with the world.

In expanding the modern resonance of classical affective ethics, Hume's sentimentalist ethics explicitly situates the roots of moral judgment in emotion rather than reason. He posits that “reason is, and ought only to be, the slave of the passions,” emphasizing that moral distinctions arise from individuals' emotional resonance with the pleasure or pain generated by actions—that is, the mechanism of “sympathy” (Hume, 2000). Within contemporary moral psychology, Jonathan Haidt's “Social Intuitionism Model” and foundational moral theories further deepen this understanding. Haidt contends that moral judgments are primarily driven by rapid, automatic emotional intuitions, with reason often providing *post-hoc* justifications for these intuitions. He categorizes universal human moral intuitions into several foundational dimensions, including “caring/harm” and “fairness/cheating,” which are highly correlated with social responsibility (Haidt, 2001). As research has deepened, academic circles have increasingly focused on the role of emotional factors in the formation of moral character in recent years (Wu, 2024). Some scholars argue that judgments about “ought” or “ought not” behaviors are often rooted in subjective feelings (Symons and VanderWeele, 2024).

In prosocial contexts such as volunteer service, participants often undergo significant emotional processes through direct engagement with social realities and deep interaction with others. However, a pressing theoretical and practical tension exists between current volunteer service practices and research paradigms, on one hand, and the fundamental requirements of education through practice in the new era and the core role of emotions in moral cultivation, on the other. On one hand, national policies explicitly position volunteer service as a key vehicle for “cultivating and practicing core socialist values” and “adhering to practice-based education,” with its core objective being to “enhance societal responsibility and dedication.” The theoretical lineage from Aristotle and Hume to Haidt also clearly reveals that the formation of stable responsible character is not achieved through simple cognitive indoctrination or mechanical repetition of behaviors. Rather, it must occur through profound and positive moral emotional experiences and internalization. On the other hand, existing research on volunteering's impact on social responsibility generally lacks a refined psychological process model that systematically explains how external volunteering practices translate into stable internal emotional character within individuals. It fails to fully reveal how, within the emotionally charged social interaction arena of volunteering, an individual's ability to recognize, understand, and manage related emotions, along with the resulting emotional interpersonal connections, as key mediating mechanisms that synergistically catalyze the elevation of moral sentiments and the consolidation of social responsibility. This theoretical black box deprives efforts to optimize volunteering's educational functions of precise theoretical grounding.

## 2 Literature review

### 2.1 Emotional experience in university students' volunteer service and social responsibility

Volunteer service refers to voluntary, non-profit, and non-professional actions in which college students dedicate their goodwill, time, skills, and resources to support public life and promote social development (Khasanzyanova, 2017). Previous studies have explored the relationship between participation in volunteer service and college students' social responsibility through theoretical deduction, individual interviews, and survey descriptions. For example, MacNeela and Gannon (2014) found through interviews that participation in volunteer service produces a progressive effect on college students' moral development. In addition, volunteer service can stimulate students' patriotism, facilitate the transformation of their social roles (Khudoykulov, 2024), and, as a form of hidden curriculum, enhance their social-emotional competence. These studies collectively suggest a certain relationship between volunteer service and college students' social responsibility. However, it remains unclear whether the act of participating in volunteer service alone is sufficient to enhance a sense of social responsibility.

Volunteer service, as a highly emotionally demanding activity, requires participants to invest not only physical and intellectual effort but also emphasizes deep emotional engagement and commitment. By actively participating in volunteer service, college students gain exposure to diverse social contexts, thereby acquiring rich emotional experiences and enhancing their emotional cognition and expression skills (Flanagan et al., 2007). During these activities, students must meticulously understand the actual needs of service recipients and build trusting relationships through effective communication. Beyond acquiring task-related practical information, students experience a two-way flow of emotional information. This interaction helps enhance their emotional intelligence and social insight. Direct engagement with service recipients activates the mirror neuron system in volunteers‘ brains, enabling them to mirror and internalize others' emotional states, thereby triggering profound empathy. Supported by this neural mechanism, volunteers become more attuned to the emotional shifts of service recipients, deepening emotional connections (Yin et al., 2025).

Based on the constructivist theory of positive emotions, the emotional experiences in volunteer service promote the internalization of social responsibility among college students through multiple pathways. Specifically, cognitive expansion helps individuals broaden their horizons and transcend self-limitations; resource construction strengthens support from personal and social networks; while meaning construction deepens reflection on life values and goals, thereby comprehensively driving the internal transformation of responsibility (Fredrickson, 2001; Xiao et al., 2025). During the broadening effect stage, positive emotions such as joy and a sense of accomplishment expand individuals' attention from a self-centered focus to societal needs, shifting their cognitive patterns from rigid to flexible and innovative. In the building effect stage, repeated accumulation of positive emotional experiences, facilitated by the development of empathy, allows individuals to perceive social needs more acutely and internalize a sense of responsibility through meaning construction. For instance, when volunteer service touches on ultimate life propositions like life and death or meaning, volunteers may experience intense emotional impacts. Such profound experiences can transform social responsibility from an externally imposed norm into an intrinsic self-identity. Ultimately, this internalization process helps form a stable and enduring responsibility-oriented personality, influencing long-term life choices and social participation.

Based on the above, the following hypotheses are proposed:

*Hypothesis 1 (H1):* University students who participate in volunteer service exhibit a stronger sense of social responsibility than those who do not.
*Hypothesis 2 (H2)*: The stronger the emotional experience in volunteer service, the stronger the social responsibility of university students.

### 2.2 The mediating role of emotional intelligence

Emotional experiences in volunteer service may also influence social responsibility by enhancing emotional intelligence. Emotional Intelligence (EI), proposed by Salovey (Yale University) and Mayer (University of New Hampshire), refers to an individual's ability to monitor one's own and others' emotions, recognize and use this information to guide thoughts and behaviors (Mayer et al., 1999, 2025). Research on EI has mainly focused on three aspects: (1) conceptualization and measurement (Li and Li, 2025); foreign scholars such as Law, Wong, and Song have classified EI into four dimensions (Law et al., 2004), while domestic researchers like Lu Jiamei have identified core psychological operations of EI, including observation, understanding, evaluation, anticipation, experience, expression, and regulation (Yang and Liu, 2024); (2) correlations between EI and other variables, such as personality, self-efficacy, psychological capital, cognitive intelligence, creativity, academic performance, occupational burnout, subjective wellbeing, and job satisfaction (Lu, 2005; Xu and Choi, 2023); (3) differences in EI levels among populations, its characteristics, and cultivation methods—for instance, significant gender and grade differences exist in college students' EI (Zhang and Xu, 2004).

Existing research demonstrates that extracurricular activities significantly enhance students' emotional cognition, emotional regulation, and interpersonal communication skills. These activities include, but are not limited to, sports competitions, artistic creation, club participation, and social practice (Guilmette et al., 2019; Feraco et al., 2023). Diverse social practices and altruistic activities such as volunteer services not only enrich students' social experiences but also effectively improve graduate students' psychological resilience, thereby further enhancing their emotional intelligence. This helps them maintain emotional stability and positive coping abilities when facing stress and challenges (Chen et al., 2023). The Expanding-Constructing Theory provides theoretical support for these findings: It suggests that through continuous accumulation of empathy skills in activities like volunteer services, students can optimize their emotional regulation strategies and strengthen self-efficacy in emotional management. This process not only helps consolidate positive emotional experiences but also gradually forms more stable social responsibility tendencies and prosocial behavior patterns, thereby establishing a virtuous cycle between personal development and social adaptation. The following hypothesis is put forward:

*Hypothesis 3 (H3):* The emotional experience of volunteer service has a significant impact on university students' emotional intelligence.

The interaction between Emotional intelligence (EI) and social responsibility manifests across multiple dimensions. EI not only profoundly influences students' comprehension of social responsibility's essence but also significantly shapes their cognitive approaches to personal obligations, as well as their willingness and effectiveness in practicing social responsibility behaviors (Guillen et al., 2022; Rasoolimanesh et al., 2023). Specifically, emotional intelligence involves an individual's ability to recognize, understand, express, and regulate their own and others‘ emotions, while social responsibility emphasizes the moral and ethical duties individuals should undertake in social relationships. Both are grounded in an understanding of the relationship between individuals and society, forming a close connection at the behavioral level.

From a cognitive perspective, emotional intelligence enhances individuals' empathy and social awareness, enabling them to better understand societal needs and others' emotional states, thereby strengthening their value identification with social responsibility. Those with high emotional intelligence can more acutely perceive social injustices, others 'hardships, or collective goals, thus more proactively combining self-actualization with social contribution. Behaviorally, emotional intelligence supports individuals in effectively managing emotional conflicts and maintaining cooperative attitudes during social interactions, promoting sustained responsible behaviors such as volunteerism, advocating for social justice, or practicing environmental protection. Therefore, social responsibility can be regarded as a key outcome variable of emotional intelligence, reflecting both its social extension of emotional capabilities and its behavioral transformation in real-world contexts. Particularly in structured social practices like volunteerism, students immerse themselves in authentic social-emotional scenarios, using emotional experiences to identify, regulate, and predict their own and others' emotional responses more effectively. This process not only strengthens their emotional regulation and interpersonal coordination skills but also deepens their understanding of “individual society” relationships, helping them recognize their responsibilities as social members and gradually establish stable patterns of social responsibility behavior. Based on the above analysis, the following hypothesis is proposed:

*Hypothesis 4 (H4):* University students' emotional intelligence has a significant impact on their sense of social responsibility.

### 2.3 The mediating role of relational embeddedness

The concept of embeddedness was first introduced by Granovetter (1973) in Economic Action and Social Structure: The Problem of Embeddedness. It refers to the embedding of economic activities within social networks formed by environmental interactions, where the elements of the network significantly influence the decision-making and actions of individuals. This theory focuses on the interpersonal connections among network participants, emphasizing the level of trust, intensity of interaction, and the quality of relationships. The concept has since been widely adopted in fields such as sociology, organizational behavior, and education to analyze how individuals act and are constrained within specific social structures.

The key distinction between volunteer service and traditional moral education lies in the fact that volunteers achieve physical and emotional immersion within the service context, rather than maintaining an external, detached moral stance (Lv et al., 2024). Traditional moral education often emphasizes the transmission of external norms and the shaping of moral images, which can lead to passive acceptance or even emotional detachment on the part of the learner. In contrast, volunteer service builds real, concrete relationships with others, fostering mutual embedding, which encourages volunteers to achieve emotional resonance and value identification. This process more effectively facilitates the growth and deepening of intrinsic moral consciousness.

In college student volunteer services, relational embeddedness refers to the depth of connections and mutual influence between volunteers. The emotional experiences generated through volunteer service dynamically facilitate the formation and deepening of relational embedding. Volunteer scenarios encompass not only interpersonal interactions but also engagements between individuals and their environment, as well as between people and tasks. Positive emotions arising from these processes—such as empathy, joy, and a sense of accomplishment—can broaden volunteers' social awareness and enhance their acceptance and openness toward team members. For instance, collective pride stemming from team achievements not only strengthens cohesion but also motivates volunteers to proactively share experiences and resources, thereby further reinforcing emotional embedding and a sense of belonging among members (Waugh and Fredrickson, 2006). Based on the above analysis, the following hypothesis is proposed:

*Hypothesis 5 (H5):* The emotional experience of volunteer service has a significant impact on college students' relational embeddedness.

Research demonstrates that relational embeddedness significantly influences the development of personal social responsibility (Casino-García et al., 2021). For instance, in volunteer work, establishing positive and stable social relationships with colleagues, service recipients, and community members can effectively motivate and sustain volunteers' responsible behaviors and ethical awareness (Gill, 2023). Moreover, changes in intimacy levels within adult relationships significantly influence the development of responsibility. Intimate and positive interactions particularly facilitate the internalization of moral identity (Yang et al., 2025).

*Hypothesis 6 (H6): The* Relational Embeddedness of college students in volunteer service has a significant impact on their sense of social responsibility.

### 2.4 The relationship between emotional intelligence and relationship embeddedness

Emotional intelligence, as the core ability for individuals to perceive, understand, and regulate their own and others' emotions, is closely associated with relational embedding in volunteer services. Existing research indicates that empathy and emotional regulation within the emotional intelligence framework show significant positive correlations with the quality of social interactions (Salovey and Mayer, 1990). Specifically, in college student volunteer scenarios, volunteers with higher emotional intelligence are more adept at accurately capturing emotional signals from service recipients or team members. Through empathetic responses, they establish emotional connections, thereby transforming weak ties into strong ones and deepening relational embedding (Paxton et al., 2020). For instance, when teams face task challenges that induce anxiety, emotionally intelligent volunteers can proactively regulate their own emotions and comfort others, fostering mutual support and trust among members, which enhances team emotional embedding. Additionally, emotional management skills within emotional intelligence help volunteers effectively resolve conflicts in cross-group interactions, maintain stable interpersonal connections, and ensure the sustained development of relational embedding. Based on this, Hypothesis 7 is proposed:

Hypothesis 7 (H7): University students' emotional intelligence has a significant positive impact on relational embedding in their volunteer services.

### 2.5 Conceptual model

**Figure 1:**
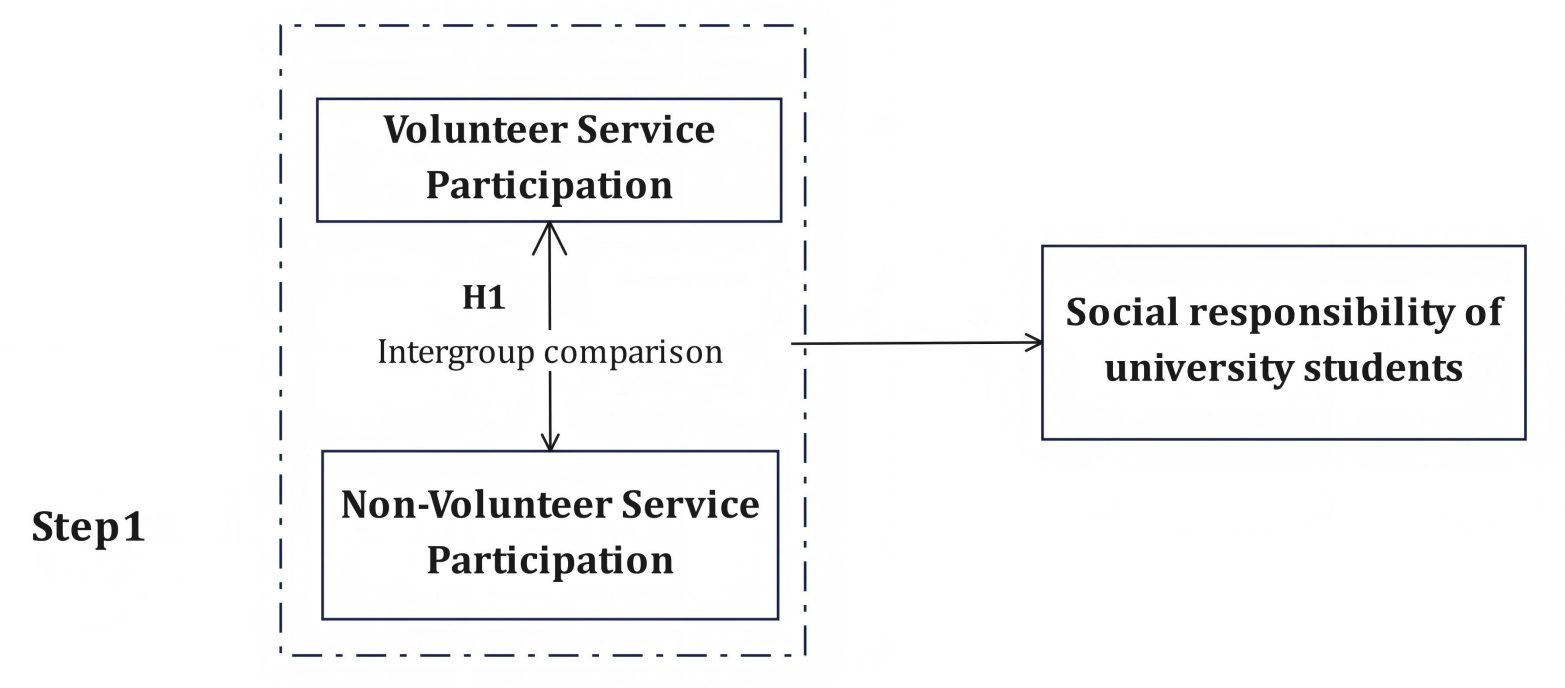
Intergroup comparison framework between volunteer and nonvolunteer university students.

**Figure 2:**
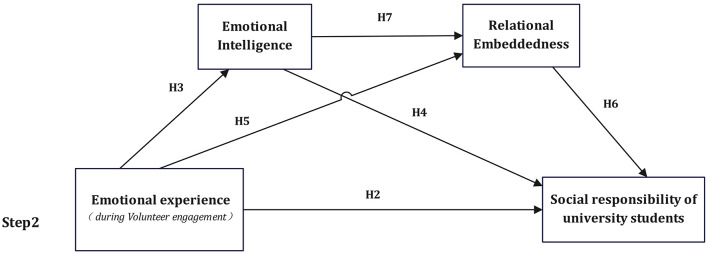
Theoretical model. H8 represents the overall indirect effect of emotional experience on social responsibility through emotional intelligence and relational embeddedness.

The primary objective of this study is to examine the impact of emotional experiences arising from volunteer service on enhancing Chinese university students‘ sense of social responsibility, and to explore the relationship between emotional experiences and students' social responsibility. Beyond the mediating effects of emotional intelligence and relational embeddedness, we further hypothesize a direct relationship between emotional intelligence and relational embeddedness:

Hypothesis 8 (H8): The relationship between university students' emotional experiences and social responsibility is mediated by MA and MB. We then developed a conceptual model that included all hypotheses (Figures 1, 2).

As illustrated in Figure 1, this study adopts a two-step analytical framework. In the first step, an intergroup comparison is conducted to examine differences in social responsibility between students who participate in volunteer service and those who do not. This comparison directly tests Hypothesis 1 (H1), which posits that university students with volunteer service experience exhibit a higher level of social responsibility than non-volunteers.

Figure 2 presents the theoretical model for students who engage in volunteer service and depicts the hypothesized relationships among emotional experience, emotional intelligence, relational embeddedness, and social responsibility. Within this model, emotional experience during volunteer engagement is treated as the core antecedent variable. A direct path from emotional experience to social responsibility is specified to test Hypothesis 2 (H2). In addition, emotional experience is hypothesized to positively influence emotional intelligence (H3) and relational embeddedness (H5).

Emotional intelligence is proposed as a key psychological mechanism that facilitates both social responsibility (H4) and relational embeddedness (H7). Relational embeddedness, in turn, is hypothesized to exert a significant positive effect on students' social responsibility (H6). Together, these paths reflect both parallel and serial mediation mechanisms, whereby emotional intelligence and relational embeddedness jointly transmit the effect of emotional experience on social responsibility.

Overall, the conceptual model integrates emotional, relational, and moral dimensions of volunteer service participation, providing a comprehensive framework for understanding how emotional experiences in volunteer contexts are translated into stable social responsibility outcomes among university students. This structure allows for the empirical testing of Hypothesis 8 (H8), which proposes that emotional intelligence and relational embeddedness mediate the relationship between emotional experience and social responsibility.

## 3 Research design

### 3.1 Research sample

This study employed a cross-sectional survey design targeting university student in China. Participants were drawn from 28 provinces (autonomous regions and municipalities), covering a wide range of academic disciplines, including sociology, education, medicine, engineering, science, and agriculture. A snowball sampling method was employed to distribute the questionnaire. In total, 5,637 questionnaires were distributed, and 5,462 were returned, yielding a response rate of 96.9%.

Following data screening, 374 questionnaires were excluded due to completion times under 1 min or exceeding 15 min, uniformly extreme responses, or participants reporting major life events within the past year. The final valid sample comprised 5,088 respondents. Among them, 4,498 students participated in volunteer service during the past year, while 590 had not.

### 3.2 Research instruments

#### 3.2.1 Measurement of emotional experience in volunteer service

The Emotional Experience Scale employs two items adapted from Plutchik (1955)'s *Emotional Experience Questionnaire* to measure the intensity of emotional experiences. Responses are rated on a 5-point Likert scale (1 = “Strongly Disagree,” 5 = “Strongly Agree”). Higher scores indicate stronger emotional experiences.

#### 3.2.2 Measurement of social responsibility

The assessment of social responsibility was conducted using a 12-item scale adapted from Liu et al. (2020). The scale, entitled the 'China College Students' Social Responsibility Scale', encompasses four dimensions: national/ethnic responsibility, helping others, environmental protection, and organizational/collective responsibility. Items were rated on a 5-point Likert scale (1 = “strongly disagree,” 5 = “strongly agree”). Higher scores indicated stronger social responsibility.

#### 3.2.3 Measurement of emotional intelligence

Emotional intelligence was measured using a 12-item scale adapted from Yongmei et al. (2011) in the Chinese context, covering four dimensions: emotional regulation, others' emotion assessment, emotion utilization, and self-emotion evaluation. Rated on a 5-point Likert scale (1 = “strongly disagree,” 5 = “strongly agree”), higher scores indicated higher emotional intelligence. Cronbach's α = 0.951.

#### 3.2.4 Measurement of relationship embeddedness

Researchers used a 15-item scale to measure relationship embeddedness. This scale was adapted from Wu et al. (2019). It included relationship strength and quality. Since students mainly interacted with two groups during volunteering (team members and service recipients), the scale measured both separately for each group. They were given a rating on a 5-point scale where 1 was “strongly disagree” and 5 was “strongly agree.” Higher scores showed a stronger connection to others.

The specific definitions, coding methods, and operationalization of the independent variables, dependent variables, mediating variables, and control variables involved in this study are detailed in Table 1. The constituent dimensions of each scale and the specific measurement items in the questionnaire survey are reported in Appendix A.

**Table 1:** The detailed descriptions of the corresponding variables.

Variable type	Variable name	Variable description
P Has the individual participated in volunteer services?		No = 0; Yes = 1
Independent Variable	X Volunteer Service Emotional Experience	Not at all = 1; Not really = 2; Somewhat = 3; Quite = 4; Very much = 5
	Y Sense of Social Responsibility	Not at all = 1; Not really = 2; Somewhat = 3; Quite = 4; Very much = 5
	*Y1 National and Ethnic Responsibility*	
Dependent Variable	*Y2 Environmental Conservation Responsibility*	
	*Y3 Responsibility to Assist Others*	
	*Y4 Organizational and Collective Responsibility*	
	MA Emotional Intelligence	Not at all = 1; Not really = 2; Somewhat = 3; Quite = 4; Very much = 5
	*MA1 Emotional Regulation Ability*	
	*MA2 Ability to Assess Others' Emotions*	
	*MA3 Ability to Utilize Emotions*	
	*MA4 Ability to Self-Assess Emotions*	
	MB Relationship Embedding	Not at all = 1; Not really = 2; Somewhat = 3; Quite = 4; Very much = 5
	*MB1 Strength of Relationship with Service Recipients*	
	*MB2 Quality of Relationship with Service Recipients*	
	*MB3 Strength of Team Relationships*	
	*MB4 Quality of Team Relationships*	
Control variables	A Gender	Male = 1; Female = 2
	B Ethnicity	Han ethnicity = 1; Ethnic minorities = 2
	C School Type	Double First-Class universities = 1; Regular undergraduate institutions = 2; Higher vocational colleges = 3
	D Grade Level	Lower-year students = 1; Upper-year students = 2
	E Political Affiliation	General public = 1; Communist Youth League members = 2; The Communist Party of China(CPC) members = 3; Others = 4
	F Family Economic Status	Excellent = 1; Good = 2; Average = 3; Difficult = 4; Very difficult = 5
	G Duration of Volunteer Service Participation	1-20 hours = 1; 21-40 hours = 2; 41-60 hours = 3; 61-80 hours = 4; 81 hours and above = 5

## 4 Data analysis

### 4.1 Comparative analysis of participation and non-participation in

Fisher's exact chi-square tests were conducted to examine group differences in control variables (gender, ethnicity, school type, grade level, political affiliation, and family economic status). As shown in Table 2, no significant differences were observed between the two groups. This indicates that the volunteer and non-volunteer groups were comparable in terms of demographic characteristics.

**Table 2:** Cross-tabulation (Chi-square) Analysis Results.

Variables	Categories	Have you participated in volunteer service? (%)		Total	χ^2^	*p*
		*Have participated in volunteer service (N = 4,498)*	*Have not participated in volunteer service (N = 590)*			
Gender	*Male*	1,352 (30.06)	190 (32.20)	1,542 (30.31)	1.137	0.286
	*Female*	3,146 (69.94)	400 (67.80)	3,546 (69.69)		
Total		4,498	590	5,088		
Ethnicity	*Han ethnicity*	3,907 (86.86)	504 (85.42)	4,411 (86.69)	0.934	0.334
	*Ethnic minorities*	591 (13.14)	86 (14.58)	677 (13.31)		
Total		4,498	590	5,088		
School Type	*Double First-Class University*	693 (15.41)	104 (17.63)	797 (15.66)	2.241	0.326
	*Regular undergraduate institutions*	3,299 (73.34)	417 (70.68)	3,716 (73.03)		
	*Higher vocational colleges*	506 (11.25)	69 (11.69)	575 (11.30)		
Total		4,498	590	5,088		
Grade Level	*Lower-year students*	3,277(72.85)	420(71.19)	3,697(72.66)	0.731	0.393
	*Upper-year students*	1,221(27.15)	170(28.81)	1,391(27.34)		
Total		4,498	590	5,088		
Political Affiliation	General public	579(12.87)	88(14.92)	667(13.11)	3.365	0.339
	*Communist Youth League members*	3,629(80.68)	469(79.49)	4,098(80.54)		
	*The Communist Party of China(CPC) members*	263(5.85)	28(4.75)	291(5.72)		
	*Others*	27(0.60)	5(0.85)	32(0.63)		
Total		4498	590	5088		
Family Economic Status	*Excellent*	191(4.25)	16(2.71)	207(4.07)	4.542	0.338
	Good	1,373(30.52)	174(29.49)	1,547(30.40)		
	Average	2,462(54.74)	340(57.63)	2,802(55.07)		
	Difficult	358(7.96)	48(8.14)	406(7.98)		
	Very difficult	114(2.53)	12(2.03)	126(2.48)		
Total		4,498	590	5,088		

Normality tests were conducted for all variables in both groups. As reported in Appendix B, absolute values of skewness and kurtosis for all variables were within acceptable thresholds (|skewness| < 3, |kurtosis| < 10), indicating no substantial deviation from normality and supporting the use of parametric tests.

Independent-samples *t*-tests were then performed to examine differences in emotional intelligence, relational embeddedness, and sense of social responsibility between volunteers and non-volunteers. As shown in Table 3, there were no significant differences between the groups for any of these variables (all *p* > 0.05). **Therefore, Hypothesis 1 was not supported**.

**Table 3:** Independent sample *t*-test analysis results.

Dependent variable	Have you participated in volunteer service? (%)		*t*	*p*
	*Have participated in volunteer service (N = 4,498)*	*Have not participated in volunteer service (N = 590)*		
Y Sense of Social Responsibility	3.98 ± 0.70	3.95 ± 0.91	0.814	0.416

^*^p < 0.1, ^**^p < 0.05, ^***^p < 0.01, ^****^p < 0.001.

### 4.2 Differential analysis of the sample participating in volunteer services (*N* = 4498)

The results of the independent samples *t*-test for university students' sense of social responsibility are shown in Table 4. Significant differences in social responsibility were found across ethnicity, school type, political affiliation, family economic status, and volunteer service duration, while gender and grade level had no significant impact.

**Table 4:** Social responsibility across demographics and volunteer service duration.

Demographics			Samples	Mean	Standard Deviation	*F*	*p*
Y Sense of Social Responsibility	Gender	*Male*	1,352	3.99	0.72	0.336	0.562
		*Female*	3,146	3.98	0.69		
		Total	4,498	3.98	0.7		
	Ethnicity	*Han ethnicity*	3,907	3.99	0.7	3.885	0.049^*^
		*Ethnic minorities*	591	3.93	0.71		
		Total	4,498	3.98	0.7		
	School Type	*Double First-Class University*	693	4.06	0.74	5.089	0.006^**^
		*Regular undergraduate institutions*	3,299	3.97	0.68		
		*Higher vocational colleges*	506	3.99	0.77		
		Total	4,498	3.98	0.7		
	Grade Level	*Lower-year students*	3,277	3.99	0.69	1.375	0.241
		*Upper-year students*	1,221	3.96	0.73		
		Total	4,498	3.98	0.7		
	Political Affiliation	General public	579	3.97	0.74	7.998	0.000^**^
		*Communist Youth League members*	3,629	3.97	0.69		
		*The Communist Party of China(CPC) members*	263	4.18	0.72		
		*Others*	27	4.14	0.66		
		Total	4,498	3.98	0.7		
	Family Economic Status	*Excellent*	191	4.15	0.77	8.988	0.000^**^
		*Good*	1,373	4.03	0.66		
		*Average*	2,462	3.95	0.71		
		*Difficult*	358	3.9	0.69		
		*Very difficult*	114	4.16	0.78		
		Total	4,498	3.98	0.7		
	Duration of Volunteer Service Participation	*1-20 hours*	217	3.99	0.54	8.252	0.000^***^
		*21-40 hours*	2,694	3.94	0.71		
		*41-60 hours*	876	4.02	0.66		
		*61-80 hours*	357	4.1	0.74		
		*81 hours and above*	354	4.11	0.76		
		Total	4,498	3.98	0.7		

^*^p < 0.1,

^**^p < 0.05,

^***^p < 0.01.

### 4.3 Structural equation modeling analysis

The Structural Equation Modeling (SEM) was used to further analyze the groups of volunteers (*N* = 4,498), with emotional experience as the independent variable, emotional intelligence and relationship embedding as the mediating variables, and social responsibility as the dependent variable.

#### 4.3.1 Common method bias test and procedural control

Although we used scales to measure the variables, all the data was collected in a short time, which could have caused common method bias (CMB). We used a lot of different ways to check the results, including pre-testing the questionnaire many times, getting people to complete the questionnaire anonymously, and using a statistical test called Harman's single-factor test. First, we analyzed whether the data were suitable for factor analysis. As shown in Table 4, the KMO value of 0.98 exceeds 0.6, meeting the prerequisite for factor analysis, indicating that the data are suitable for such research. Additionally, the data passed the Bartlett's test for sphericity (*p* < 0.05), further confirming their suitability for factor analysis. The results of an exploratory factor analysis (EFA) revealed that the first factor accounted for 41.79% of the variance, with a cumulative variance explained of 61.62% (Appendix C).

#### 4.3.2 Reliability and validity testing

Reliability and validity assessments were conducted using SPSS 26.0 and AMOS 26.0 software for the scales measuring emotional experience and its subscales, emotional intelligence and its subscales, relational embeddedness and its subscales, and social responsibility and its subscales. Cronbach's α coefficients for all subscales exceeded 0.9, indicating strong reliability. Detailed reliability and validity results are presented in (Appendix D). Confirmatory factor analysis (CFA) revealed that the extracted average variance extracted (AVE) values all exceeded 0.5, demonstrating good convergent validity. The square roots of AVE values were all greater than 0.722, surpassing the correlation coefficients between variables, confirming good discriminant validity.

The model demonstrated an acceptable fit. Specifically, χ^2^(774) = 10,591.228, *p* < 0.001, χ^2^/df = 13.684; GFI = 0.869, AGFI = 0.854, CFI = 0.922, IFI = 0.922, NFI = 0.917, TLI/NNFI = 0.918; RMSEA = 0.053 (90% CI: 0.047-0.056), RMR = 0.032, SRMR = 0.035; PGFI = 0.781, PNFI = 0.865, PCFI = 0.870. Although χ^2^/df exceeds conventional thresholds, this is expected given the large sample size and model complexity. Other indices indicate that the model fits the data well (see Appendix E, Table E1).

#### 4.3.3 Descriptive and correlation analysis

Table 5 reports the descriptive statistics, correlation coefficients, and variance inflation factors (VIF) values for the volunteer sample (N = 4,498). The means of the core variables ranged from 1.131 to 4.027, with standard deviations between 0.338 and 0.988, indicating moderate variability. Correlation analysis showed that Volunteer Service Emotional Experience (X), Emotional Intelligence (MA), Relational Embeddedness (MB), and Sense of Social Responsibility (Y) were positively associated (*p* < 0.01). In addition, X and Y were correlated with ethnicity, and MA and Y were correlated with family economic status (*p* < 0.01). All VIF values were below 3, suggesting that multicollinearity is unlikely to affect the analyses. Taking together, these results indicate consistent associations among emotional experience, emotional intelligence, relational embeddedness, and social responsibility, providing a basis for subsequent mediation analyses.

**Table 5:** Correlation coefficients and VIFs.

Variable	1	2	3	4	5	6	7	8	9	10	11
Mean	1.699	1.131	1.958	1.271	1.942	2.74	2.541	4.027	3.906	3.904	3.985
Standard deviation	0.459	0.338	0.515	0.445	0.456	0.767	0.988	0.926	0.72	0.723	0.701
VIF-value	1.008	1.016	1.066	1.213	1.066	1.033	1.028	1.174	1.634	1.743	1.849
1. Gender	1										
2. Ethnicity	0.027	1									
3. School type	0.023	0.011	1								
4. Grade level	−0.045^**^	0.032^*^	0.018	1							
5. Political affiliation	0.013	0.054^**^	−0.145^**^	0.167^**^	1						
6. Family economic status	0.033^*^	0.079^**^	0.139^**^	0.043^**^	−0.022	1					
7. Duration of volunteer service participation	−0.008	0.048^**^	−0.083^**^	0.008	0.068^**^	−0.019	1				
8. X: Volunteer service emotional experience	0.024	0.044^**^	0.014	0.007	0.059^**^	0.019	0.119^**^	1			
9. MA: emotional intelligence	−0.038^*^	−0.018	−0.017	0.002	0.038^*^	−0.037^*^	0.031^*^	0.256^**^	1		
10. MB: relational embeddedness	−0.017	−0.006	−0.026	−0.003	0.048^**^	−0.02	0.052^**^	0.292^**^	0.546^**^	1	
11. Y: Sense of social responsibility	−0.009	−0.029^*^	−0.030^*^	−0.017	0.047^**^	−0.050^**^	0.078^**^	0.344^**^	0.561^**^	0.596^**^	1

Have participated in volunteer service (*N* = 4,498).

^*^p < 0.1,

^**^p < 0.05,

^***^p < 0.01.

### 4.4 Mediation path analysis

To test the proposed hypotheses, this study employed Model 6 of the mediation process proposed by Hayes (2017) using the PROCESS macro (version 3.5) for SPSS. A bootstrap procedure with 5,000 resamples was applied to estimate the indirect effects, and 95% bias-corrected confidence intervals were generated. This approach does not rely on the assumption of normality and provides higher statistical power than traditional methods such as stepwise regression and the Sobel test. The results are reported in Tables 6, 7.

**Table 6:** Bootstrapped path coefficients for mediation analysis.

Hypothesis	Path	β	*P*	*Conclusion*	95% CI (LLCI, ULCI)	*R* ^2^
H2	X → Y (total effect)	0.343	^***^	*Supported*	(0.098, 0.133)	0.126
H3	X → MA	0.258	^***^	*Supported*	(0.178, 0.223)	0.069
H4	MA → Y	0.314	^***^	*Supported*	(0.280, 0.331)	0.458
H5	X → MB	0.161	^***^	*Supported*	(0.106, 0.146)	0.323
H6	MB → Y	0.376	^***^	*Supported*	(0.339, 0.390)	0.458
H7	MA → MB	0.504	^***^	*Supported*	(0.481, 0.531)	0.323
H8	X → Y (direct effect with mediators)	0.152	^***^	*Supported*	(0.239, 0.280)	0.458

^***^p-value < 0.001, ^**^p-value < 0.01, ^*^p-value < 0.05.

**Table 7:** Bootstrapped indirect effects and proportion of total effect.

Mediation path	Indirect effect (β)	95% CI (BootLLCI, BootULCI)	Conclusion	Proportion of total effect	Type of mediation
X → MA → MB → Y	0.115	(0.041, 0.058)	*Supported*	33.50%	*Chain mediation*
X → MA → Y	0.2	(0.068, 0.096)	*Supported*	58.30%	*Parallel mediation*
X → MB → Y	0.126	(0.048, 0.073)	*Supported*	36.70%	*Parallel mediation*

“Supported” indicates that the 95% bootstrap CI does not include zero.

When the mediating variables were not included, X exerted a significant positive effect on Y (β = 0.343, *p* < 0.001), supporting Hypothesis 2. In addition, X had a significant positive effect on MA (β = 0.258, *p* < 0.001), providing support for Hypothesis 3. MA was also found to have a significant effect on MB (β = 0.504, *p* < 0.001), thereby supporting Hypothesis 7. Furthermore, both MA (β = 0.314, *p* < 0.001) and MB (β = 0.376, *p* < 0.001) had significant positive effects on Y, supporting Hypotheses 4 and 6, respectively.

After simultaneously including MA and MB as mediators, the direct effect of X on Y remained significant (β = 0.152, *p* < 0.01), but its magnitude was substantially reduced compared to the total effect (β = 0.343). This result indicates the presence of partial mediation, thus supporting Hypothesis 8.

Bootstrap analyses of indirect effects further revealed that the indirect effect of X on Y through MA and MB in sequence was significant (β = 0.115), as the 95% bootstrap confidence interval did not include zero. In addition, the indirect effects through MA alone and MB alone were also significant, indicating the coexistence of both chain mediation and parallel mediation effects in the proposed model.

## 5 Result and discussion

Based on the preceding analysis, several key findings emerge:

An independent samples test revealed no significant difference in social responsibility between students who participated in volunteer work and those who did not, indicating that participation alone is insufficient to enhance social responsibility.

Students' emotional experiences during volunteering showed a strong positive correlation with social responsibility. Notably, stronger emotional experiences corresponded to higher levels of social responsibility, highlighting the critical role of emotional quality over mere behavioral participation.

Emotional intelligence and relational embeddedness significantly mediated the relationship between emotional experiences and social responsibility in college student volunteering. Additionally, emotional experiences and social responsibility were influenced by a chained mediating effect through emotional intelligence and relational embeddedness.

Overall, the findings reveal that the key driver of enhanced social responsibility among college students is not volunteer participation itself, but rather the interaction among emotional experiences, emotional intelligence, and relational embeddedness. To consolidate these research findings, Table 6 presents the results of hypothesis testing.

## 6 Conclusions and implications

### 6.1 Theoretical interpretation of research findings

This study not only supplements the “emotional-cognitive-connection” integrated framework for exploring the relationship between volunteerism and college students' sense of social responsibility but also expands the practical applications of the Emotion Extension Construction Theory.

The findings reveal that pure volunteer participation behavior shows no significant correlation with social responsibility. While this result may appear to contradict traditional cognitive theories, it highlights the essential difference between “participation forms” and “internal transformation.” Previous research has emphasized the behavioral aspects of volunteer engagement, neglecting the core role of emotional experiences. Our study further confirms that rich emotional experiences in volunteer activities can activate the emotional motivation system. Without deep emotional arousal, mere repetitive participation cannot enhance responsibility awareness. This validates the core tenets of the Emotion Extension Construction Theory: social-emotional development depends not only on behavioral participation but also on emotional experiences during activities (Fredrickson, 2001; Jackson et al., 2024). Only when volunteer activities trigger intense positive emotional experiences can social responsibility be elevated through dual pathways of emotional intelligence and relational embedding.

During the development of social responsibility, the heightened emotional engagement in volunteer work stimulates college students to develop higher emotional intelligence, thereby fostering a stronger sense of social responsibility. Understanding social relationships requires emotional intelligence (EQ) as its foundation. EQ enables individuals to discern the moral implications behind their own and others' emotions and cognitive emotions, regulate emotional intensity to promote prosocial behaviors, and facilitate more appropriate moral judgments—such as the conviction that “I ought to offer help.”

Research demonstrates a significant positive correlation between emotional experiences and relational engagement, with both factors collectively enhancing social responsibility. As a quintessential relational practice, volunteer work immerses students in a multidimensional “relational domain” that encompasses both horizontal interactions with team members and vertical emotional bonds with service recipients (Laud et al., 2015). These findings validate the application of the Emotion Extension Construction Theory in volunteer activities, thereby strengthening its explanatory power.

### 6.2 Practical recommendations

#### 6.2.1 Optimizing emotional experience design in volunteer services

Universities should shift from task-oriented activity models to emotionally immersive volunteer service designs. For instance, developing long-term, immersive programs such as rural teaching partnerships and community senior companionship initiatives allows students to actively address beneficiaries' needs, rather than engaging in short-term taken participation. Additionally, incorporating post-activity emotional reflection sessions helps students process their volunteer experiences, including moments of inspiration and empathy, thereby strengthening emotional connections.

#### 6.2.2 Integrating emotional intelligence development into volunteer service practices

Incorporate emotional management training into the volunteer service framework. For instance, conduct empathy-building and emotional regulation workshops before activities to help students cope with setbacks; provide on-site emotional support during service; and enhance students' understanding of emotional value through post-service case analyses. Additionally, universities may offer elective courses on volunteerism and emotional intelligence to bridge theory and practice.

#### 6.2.3 Enhancing relationship embedding in volunteer services

Establish a dual-dimensional connection mechanism between “teams” and “service recipients.” Teams should regularly organize collaborative tasks and team-building activities to strengthen trust and foster a sense of belonging among members. For service recipients, implement long-term pairing through “one-to-one” or “group-to-group” arrangements, encouraging students to maintain continuous interactions and deepen social connections. Volunteer organizations should also establish relationship tracking systems to record the frequency and quality of student interactions, serving as key evaluation metrics for activities. The suggestions are aimed at solving the dilemma of “formalism and lack of effectiveness” of volunteer service from the three core dimensions of emotional experience, ability cultivation and social connection, and realizing the value of volunteer service in improving the sense of social responsibility of college students.

### 6.3 Limitations and future directions

The following aspects need to be improved, and the following research directions should be improved.

First, sample representativeness is limited. Factors such as participants' nationalities and the types of their affiliated institutions were not considered, and the generalizability of the conclusions requires further validation. Future studies could employ multi-stage stratified sampling to expand the sample coverage to countries at different developmental stages, incorporating and integrating several types of institutions, including comprehensive, science and engineering, and humanities, to enhance the external validity of the conclusions.

Secondly, the cross-sectional data collection method is employed. This approach struggles to capture dynamic evolutionary processes between variables. The impact of volunteerism on college students' sense of social responsibility shows long-term cumulative effects, while single-point measurements cannot reveal their developmental trajectory or the stability of causal relationships. Future research should adopt longitudinal tracking designs, conducting annual follow-up surveys to document the temporal changes in volunteer participation duration and students‘ sense of social responsibility. This approach would better prove long-term effects and the stability of causal relationships.

Third, variable measurements could be further refined. The study did not differentiate between types of volunteer services, such as educational assistance, community service, and environmental public welfare, which may obscure the heterogeneity of emotional experiences across activities and their differential impacts on mediating variables. Future research could conduct grouped comparisons by service type to analyze the correlation strength between empathy capacity and social responsibility in educational assistance activities, the unique mechanisms of relational embedding in community service, and the distinct characteristics of value arousal effects in environmental public welfare activities.

Fourth, the exploration of medium-sized mechanisms remains insufficient. While the study confirmed the parallel mediating role of emotional intelligence and relationship embeddedness, it failed to examine their interaction effects or incorporate potential variables such as self-efficacy. The complexity of mediating pathways requires further investigation. Future research could develop moderated mediation models to test the interaction between emotional intelligence and relationship embeddedness, while introducing variables like self-efficacy and empathy to explore chain or parallel multiple mediation mechanisms.

Finally, the moderating role of individual traits was not adequately considered. While the study controlled for variables such as gender and grade level, the absence of personality traits like neuroticism and openness, along with factors related to growth experiences, may have overlooked individual differences in how emotional experiences translate into social responsibility. Future research could incorporate the neuroticism and openness dimensions from the Big Five Personality Inventory, combined with variables like family socioeconomic status and duration of volunteer service, to analyze their moderating effects on the pathway from emotional experiences to social responsibility.

## Data Availability

The datasets presented in this article are not readily available because they are part of an ongoing doctoral dissertation conducted by the first author, and the dissertation has not yet been publicly released. Requests to access the datasets should be directed to the corresponding author, LO, at oulingjunla@163.com.
