# Supplementary figures and images for: How volunteer engagement experiences relate to the social responsibility of university students: the roles of emotional intelligence and relational embeddedness
Source: Front Psychol. 2026 Apr 29;17:1759354. doi: 10.3389/fpsyg.2026.1759354 (PMC13168824; doi:10.3389/fpsyg.2026.1759354)

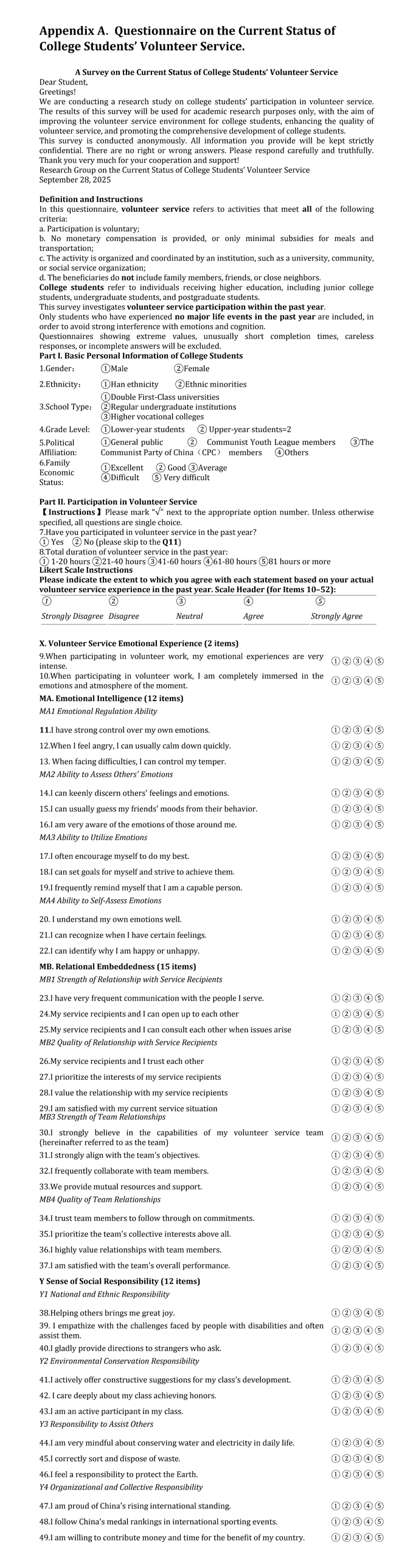

Supplement: Supplementary file 1 [file Data_Sheet_1.zip › Supplementary material presentation/Appendix A. Questionnaire on the Current Status of College Students’ Volunteer Service..jpg]

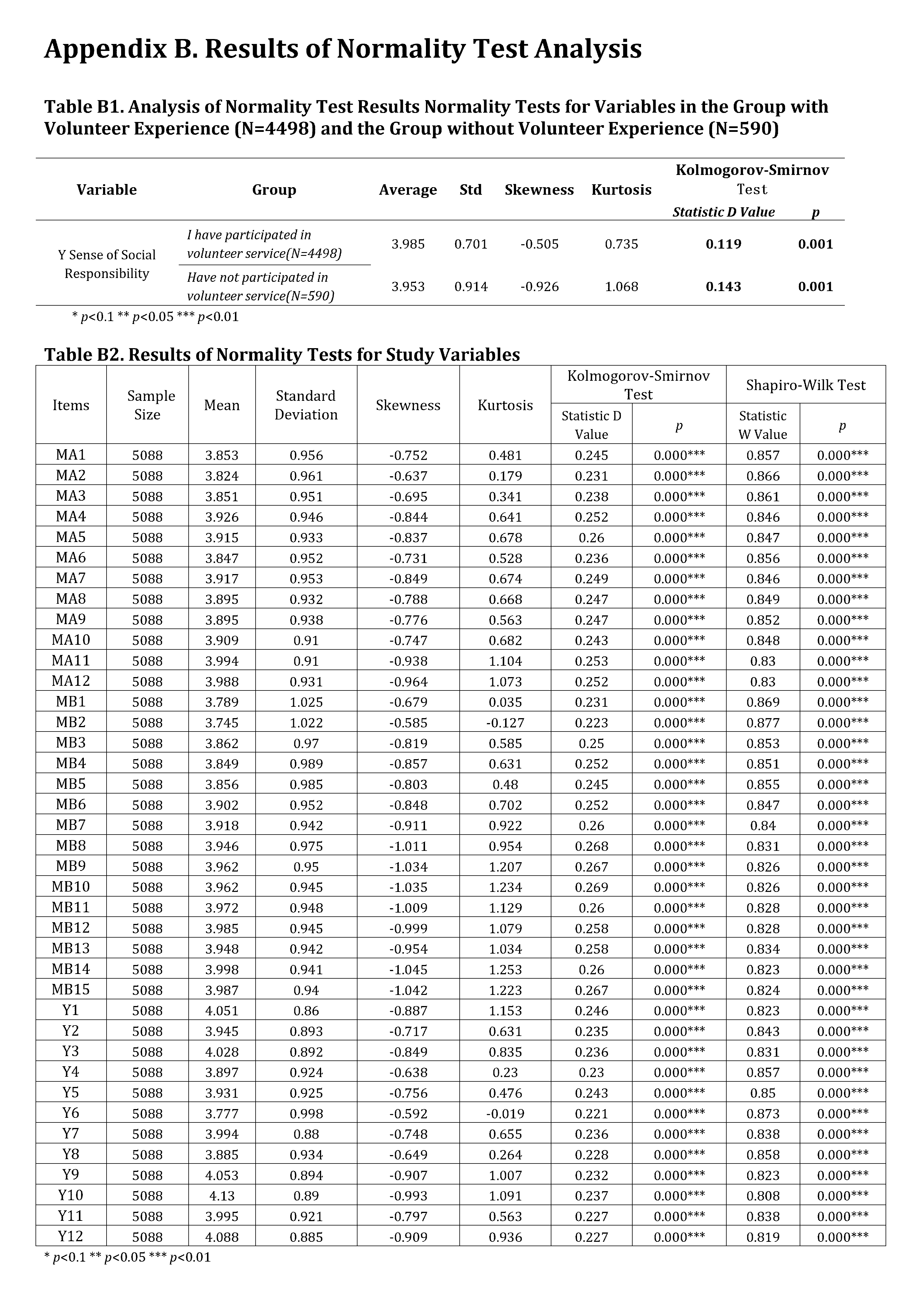

Supplement: Supplementary file 1 [file Data_Sheet_1.zip › Supplementary material presentation/Appendix B. Results of Normality Test Analysis.jpg]

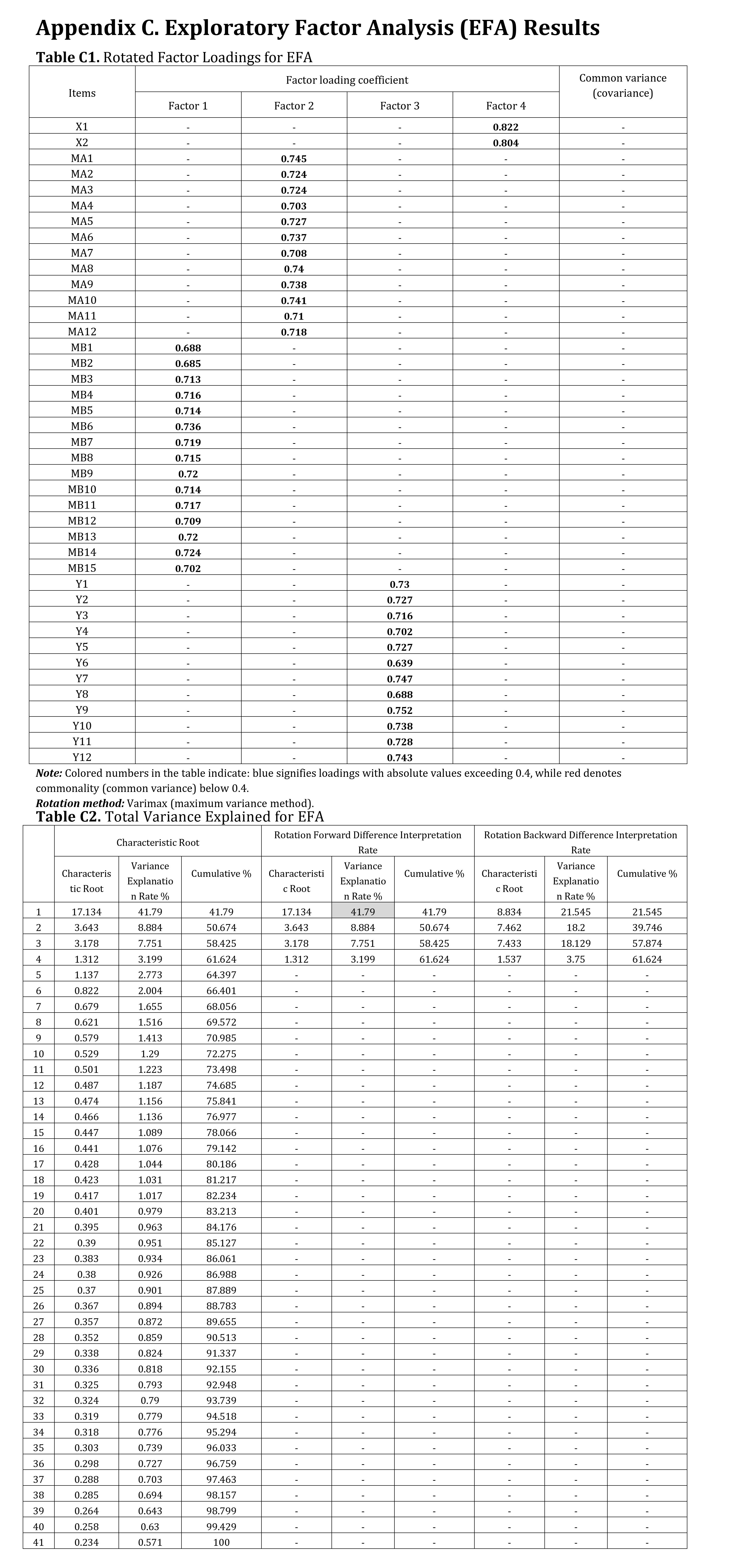

Supplement: Supplementary file 1 [file Data_Sheet_1.zip › Supplementary material presentation/Appendix C. Exploratory Factor Analysis (EFA) Results.jpg]

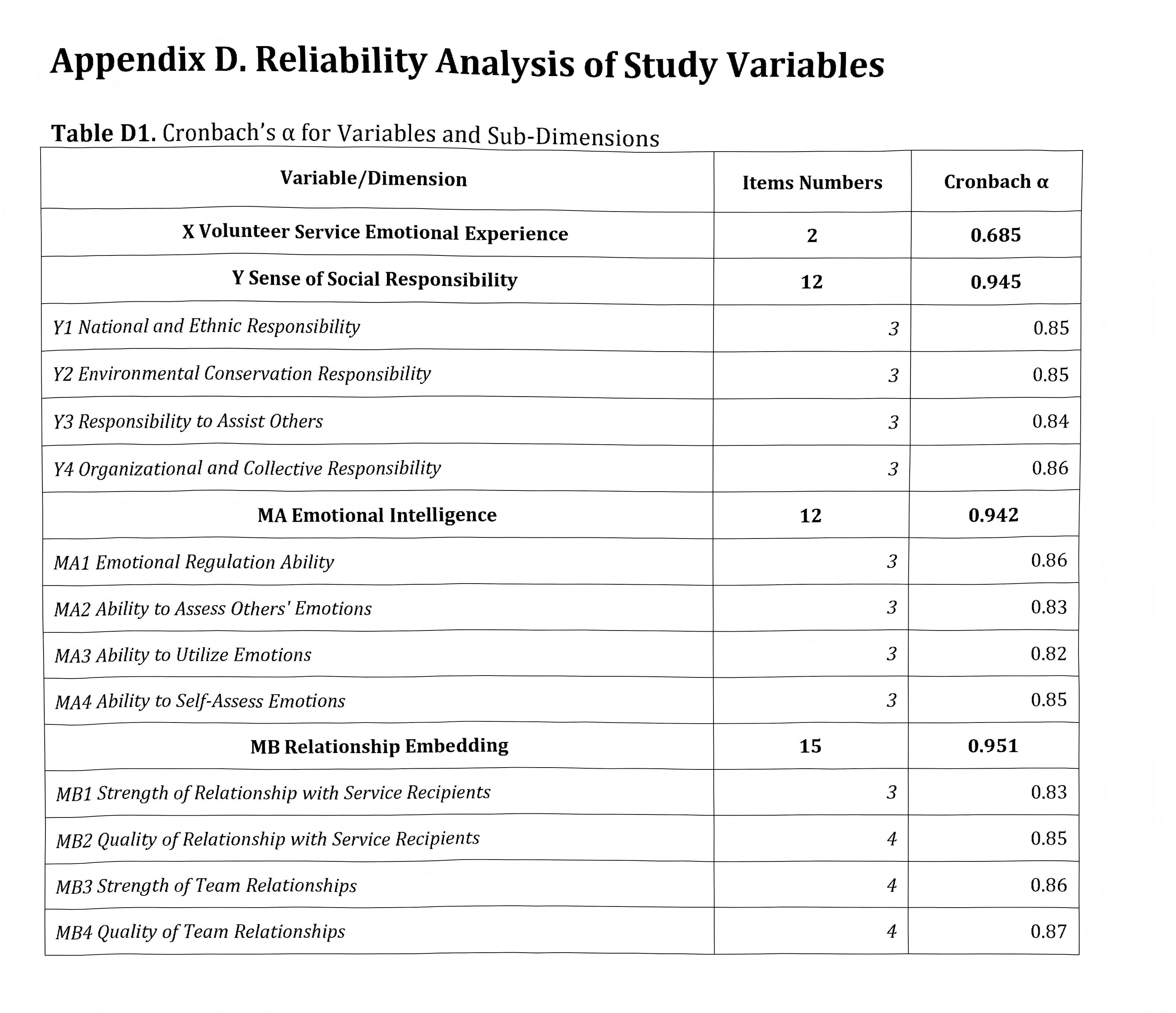

Supplement: Supplementary file 1 [file Data_Sheet_1.zip › Supplementary material presentation/Appendix D. Reliability Analysis of Study Variables.jpg]

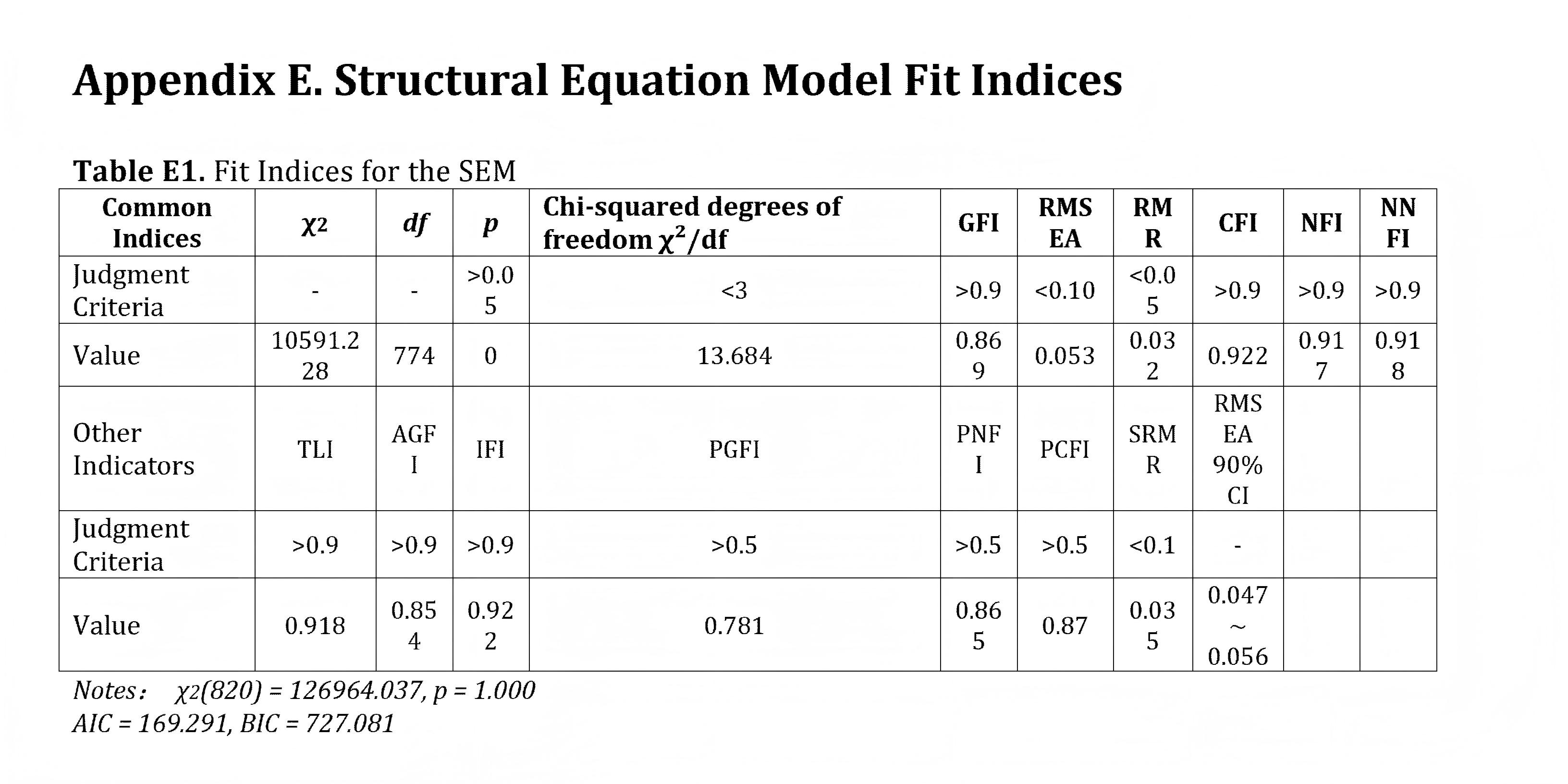

Supplement: Supplementary file 1 [file Data_Sheet_1.zip › Supplementary material presentation/Appendix E. Structural Equation Model Fit Indices.jpg]
